# Supplementary material for: Metabolites Potentially Determine the High Antioxidant Properties of Limosilactobacillus fermentum U-21
Source: BioTech (Basel). 2023 May 17;12(2):39. doi: 10.3390/biotech12020039 (PMC10204573; doi:10.3390/biotech12020039)
Supplement: Supplementary file 1 [file biotech-12-00039-s001.zip › Supplementary.pdf]

**Table S1.** Main classes of common metabolites to *L.fermentum* U-21 *and L.fermentum* 279 detected in the aqueous phase of culture supernatant.

| Metabolite | Class |
|---|---|
| Glycine (Gly) | Amino acids and derivatives |
| Alanine (Ala) | |
| Valine (Val) | |
| Leucine (Leu) | |
| Isoleucine (Ile) | |
| Aspartic acid (Asp) | |
| Asparagine (Asn) | |
| Glutamic acid (Glu) | |
| Glutamine (Gln) | |
| Serine (Ser) | |
| Threonine (Thr) | |
| Methionine (Met) | |
| Lysine (Lys) | |
| Histidine (His) | |
| Proline (Pro) | |
| Phenylalanine (Phe) | |
| Tyrosine (Tyr) | |
| Tryptophan (Trp) | |
| 2-Aminocaprylic acid | |
| Norleucine | |
| Homocysteine | |
| Homoserine | |
| Norvaline | |
| Ornithine | |
| N-Methyl-a-aminoisobutyric acid | |
| β-Alanine | |
| 5-Hydroxytryptophan | |
| Pyroglutamic acid | |
| Aceturic acid | |
| a-Methyl-L-tyrosine | |
| N,N-Dimethylglycine | |
| N-Acetyl-L-glutamic acid | |
| Carnitine | |
| Propanoic acid | Organic acid |
| Cinnamic acid | |
| Succinic acid | |
| Fumaric acid | |
| Formic acid | |
| Maleic acid | |
| Malonic acid | |
| Phthalic acid | |
| Propanedioic acid | |
| Oxalic acid | |
| 2-Aminobutanoic acid | |
| 2-Hydroxybutyric acid | |
| 3-Hydroxybutyric acid | |
| Glycolic acid | |
| Lactic Acid | |
| Tartaric acid | |

| | |
|---|---|
| Methylcitric acid | |
| Maltose | |
| Melibiose | |
| Mannobiose | |
| Cellobiose | |
| Lactose | |
| Turanose | |
| Erythrose | |
| Xylose | |
| Galactose | |
| Glucose | |
| Mannose | |
| Tagatose | |
| Fructose | |
| 2-Keto-gluconic acid | |
| Gluconic acid | |
| Mannonic acid | Saccharides |
| Ribonic acid | |
| Xylonic acid | |
| D-Erythro-Pentitol | |
| Mannitol | |
| Glycerol | |
| meso-Erythritol | |
| Arabinitol | |
| Erythritol | |
| Arabitol | |
| Arabinofuranose | |
| Dihydroxyacetone | |
| Levoglucosan | |
| Myo-Inositol | |
| Trehalose-6-phosphate | |
| Mannopyranose | |
| 2-Hydroxyisocaproic acid | |
| 4-Hydroxybutanoic acid | Fatty acids and analogues |
| Butanoic acid | |

**Table S2.** Main classes of shared metabolites of *L.fermentum* U-21 *and L.fermentum* 279 detected in the lipid-containing phase of culture supernatant.

| Metabolite | Class |
|---|---|
| Alanine (Ala) | |
| Asparagine (Asn) | |
| Proline (Pro) | |
| Tryptophan (Trp) | Amino acids and derivatives |
| 1-Aminocyclopentanecarboxylic | |
| Pyroglutamic acid | |
| Oxalic acid | |
| Phthalic acid | |
| Ritalinic acid | Organic acids |
| 4-Hydroxybenzoic acid | |
| Lyxose | Saccharides |
| Heptadecanoic acid | |
| Undecanoic acid | Fatty acids and analogues |
| Butanoic acid | |

| |
|---|
| Oleic acid |

**Table S3.** Main classes of *L.fermentum* 279 metabolites detected in the aqueous phase of culture supernatant.

| **Metabolite**[1] | **Class** |
|---|---|
| Glycine (Gly) | Amino acids and derivatives |
| Alanine (Ala) | |
| Valine (Val) | |
| Leucine (Leu) | |
| Isoleucine (Ile) | |
| Aspartic acid (Asp) | |
| Asparagine (Asn) | |
| Glutamic acid (Glu) | |
| Glutamine (Gln) | |
| Serine (Ser) | |
| Threonine (Thr) | |
| Methionine (Met) | |
| Lysine (Lys) | |
| Histidine (His) | |
| Proline (Pro) | |
| Phenylalanine (Phe) | |
| Tyrosine (Tyr) | |
| Tryptophan (Trp) | |
| 2-Aminocaprylic acid | |
| <u>5-Aminovaleric acid</u> | |
| <u>6-Aminocaproic acid</u> | |
| <u>Cystathionine</u> | |
| <u>7-Azatryptophan</u> | |
| Norleucine | |
| Homocysteine | |
| Homoserine | |
| Norvaline | |
| Ornithine | |
| N-Methyl-a-aminoisobutyric acid | |
| β-Alanine | |
| 5-Hydroxytryptophan | |
| Pyroglutamic acid | |
| Aceturic acid | |
| a-Methyl-L-tyrosine | |
| N,N-Dimethylglycine | |
| N-Acetyl-L-glutamic acid | |
| <u>N-a-Acetyl-L-Lysine</u> | |
| <u>N-Methyl-L-glutamic acid</u> | |
| <u>N-methyltryptophan</u> | |
| <u>Tranexamic acid</u> | |
| Carnitine | |
| <u>Aconitic acid</u> | Organic acid |
| <u>Acrylic acid</u> | |
| <u>Citric acid</u> | |
| <u>Ethylphosphonic acid</u> | |
| <u>β-Hydroxypyruvic acid</u> | |
| Propanoic acid | |
| Cinnamic acid | |
| Succinic acid | |

| | |
|---|---|
| Formic acid | |
| Fumaric acid | |
| Maleic acid | |
| Malonic acid | |
| Phthalic acid | |
| Propanedioic acid | |
| Oxalic acid | |
| 2-Aminobutanoic acid | |
| 2-Hydroxybutyric acid | |
| 3-Hydroxybutyric acid | |
| Glycolic acid | |
| Lactic Acid | |
| Tartaric acid | |
| Methylcitric acid | |
| Malic acid | |
| Allofuranose | Saccharides |
| Allose | |
| Arabinose | |
| Maltose | |
| Melibiose | |
| Mannobiose | |
| Cellobiose | |
| Lactose | |
| Turanose | |
| Erythrose | |
| Xylose | |
| Galactose | |
| Glucose | |
| Mannose | |
| Tagatose | |
| Fructose | |
| Rhamnose | |
| Tagatofuranose | |
| Talofuranose | |
| D-Trehalose | |
| Sucrose | |
| 2-Keto-gluconic acid | |
| Gluconic acid | |
| Mannonic acid | |
| Ribonic acid | |
| Xylonic acid | |
| Glyceraldehyde | |
| Galactopyranose | |
| Galactose oxime | |
| Glucopyranose | |
| β-D-Galactofuranose | |
| D-Glucitol | |
| Dulcitol | |
| Xylitol | |
| D-Erythro-Pentitol | |
| Mannitol | |
| Glycerol | |
| meso-Erythritol | |

| | |
|---|---|
| Arabinitol | |
| Erythritol | |
| Arabitol | |
| Arabinofuranose | |
| Dihydroxyacetone | |
| Levoglucosan | |
| Myo-Inositol | |
| Trehalose-6-phosphate | |
| Mannopyranose | |
| Methyl galactoside | |
| 2-Hydroxyisocaproic acid | |
| 4-Hydroxybutanoic acid | |
| Butanoic acid | Fatty acids and analogues |
| Decanoic acid | |
| Tiglic acid | |

[1]Underline metabolites are unique for *L. fermentum* 279

**Table S4.** Main classes of *L.fermentum* 279 metabolite detected in the lipid-containing phase of culture supernatant.

| Metabolite | Class |
|---|---|
| Alanine (Ala) | |
| Asparagine (Asn) | |
| Proline (Pro) | |
| Histidine (His) | |
| Tryptophan (Trp) | Amino acids and derivatives |
| 1-Aminocyclopentanecarboxylic | |
| Pyroglutamic acid | |
| L-Norleucine | |
| Cinnamic acid | |
| Oxalic acid | |
| Phthalic acid | |
| Ritalinic acid | Organic acids |
| 4-Hydroxybenzoic acid | |
| Toluic acid | |
| L-Sorbofuranose | |
| β-Arabinopyranose | Saccharides |
| Lyxose | |
| Arachidic acid | |
| Butanoic acid | |
| Pentadecanoic acid | |
| Propanoic acid | |
| Hexanoic acid | Fatty acids and analogues |
| Heptadecanoic acid | |
| Undecanoic acid | |
| Oleic acid | |

**Table S5.** All shared metabolites of *L.fermentum* U-21 *and L.fermentum* 279 detected in the extracellular vesicles.

| Metabolite | Class |
|---|---|
| Tryptophan (Trp) | |
| Phenylalanine (Phe) | Amino acids and derivatives |
| Alanine (Ala) | |
| Proline (Pro) | |

| | |
|---|---|
| Valine (Val) | |
| Aspartic acid (Asp) | |
| Asparagine (Asn) | |
| 3-Amino-2-piperidone | |
| 3-Methylpiperazine-2,5-dione | |
| Amino levulinic acid | |
| Norleucine | |
| L-2-Aminobutyric acid | |
| N-Methyl-a-aminoisobutyric acid | |
| Pyroglutamic acid | |
| Penicillamine | |
| Pyrrolidine-2-carboxamide | |
| Timonacic | |
| Tranexamic acid | |
| l-Norvaline | |
| 2-Aminocaprylic acid | |
| Creatinine | |
| 7-Azatryptophan | |
| Etidocaine | |
| Octadecanamide | |
| Oleamide | |
| Mercaptoacetic acid | |
| Methoxyacetic acid | |
| Glutarate | |
| Benzeneethanamine | |
| Acetic anhydride | Organic acid |
| Fumaric acid | |
| Pyrrole-2,5-dicarboxylic acid | |
| Succinic acid | |
| Pyrrole-2-carboxylic acid | |
| Citric acid | |
| Tricarballylic acid | |
| Glucosamine | |
| Ribono-1,4-lactone | |
| Fucose | |
| 3-$\alpha$-Mannobiose | |
| Lactose | |
| Turanose | |
| Xylopyranose | |
| Galactose | |
| Lyxose | |
| Glucose | |
| Erythrose | Saccharides |
| Gulose | |
| Rhamnose | |
| Glyceraldehyde | |
| Arabinonic acid | |
| Gluconic acid | |
| Erythro-Pentitol | |
| 1-Deoxypentitol | |
| Pentitol | |
| Erythritol | |
| Maltitol | |

| | |
|---|---|
| Levoglucosan | Fatty acids and analogues |
| Furan | |
| 2-Hydroxyisocaproic acid | |
| 6-Aminocaproic acid | |
| Itaconic acid | |
| 2-Hydroxy-3-methylbutyric acid | |
| 4-Pentenoic acid | |
| 1-Decanol | |
| 2-Undecen-4-ol | |
| Dodecanamide | |
| (Z)-Docos-13-enamide | |
| Hexanoic acid | |
| Propanoic acid | |
| Erucic acid | |
| Pentanamide | |
| Glycylglycine | |
| Valylvaline | |
| 1-(benzo[d][1,3]dioxol-5-yl)-2-(4-methylpiperidin-1-yl)pentan-1-one | Other organic compound |
| 1,2,4-Butanetriol | |
| 1,2-Bis(4-methoxyphenyl)ethane-1,2-diamine | |
| 1,3-Cyclohexanebis(methylamine) | |
| 1,3-Dioxolane | |
| 1,3-Propanediol | |
| 1,2,3-Butanetriol | |
| 1,2-Benzenediol | |
| (R)-(-)-2-Pyrrolidinemethanol | |
| 1,3-bis[(Trimethylsilyl)ethynyl]benzene | |
| 1,3-Dioxolane | |
| 1,3-Dioxolane-2-methanol | |
| 1,4-Bis[2-[N-[6-methoxy-8-quinolyl]amino]propionyl]piperazine | |
| 1,4-diazabicyclo[4.3.0]nonan-2,5-dione | |
| 1,6-Bis(trimethylsilyl)-1,3,5-hexatriyne | |
| 1-[2,4-Bis(trimethylsiloxy)phenyl]-2-[(4-trimethylsiloxy)phenyl]propan-1-one | |
| 17a-Aza-D-homoandrostan-17-one | |
| 1-butanamine | |
| 1-Cyclohexyldimethylsilyloxy-4-methylpentane | |
| 1-Dichloromethyl(dimethyl)silyloxyoctadecane | |
| Carbonic acid | |
| Disulfide | |
| 1-Isobutylsulfanylmethyl-2,8,9-trioxa-5-aza-1-sila-bicyclo[3.3.3]undecane | |
| 2-(1-Amino-2-1H-imidazol-1-ylethyl)-4-amino-6-dimethylamino-S-triazine | |
| 2-(2,5-Dimethoxyphenyl)-7-methoxy-2,4,6-cycloheptatrien-1-one | |
| 2-(2-Benzyldecahydroisoquinolin-3-yl)ethanol | |
| 2-(4'-Methoxyphenyl)-2-(3'-methyl-4'methoxyphenyl)propane | |
| 2-(4-Methyl-1,3-thiazol-5-yl)ethanamine | |
| 2,3,4-Trimethoxymandelic acid | |
| 2,4,4-Trimethyl-1-pentanol | |
| 2,4-Pentadienenitrile | |
| 2-[[2-[Dimethylamino]propyl]amino]-4-[trichloromethyl]-6-[a,a,a-trichloro-p-tolyl]-S-triazine | |

| |
|---|
| 2-[2-[2-Methoxyethoxy]ethoxy-1,3-dioxalane |
| 2-Cyano-5-(4-fluorophenyl)pyrimidine |
| 2-Ethyl-3-ketovalerate |
| 2--Fluorobenzylamine |
| 2-Morpholinomethyl-1,3-diphenyl-2-propanol |
| 2-Propanone |
| 2-t-Butyl-5-methyl-[1,3]dioxolane-4-carboxylic acid |
| 2-Undecanethiol |
| 3,5-Di(2-thienyl)pyridine |
| 3,6-Dioxa-2,4,5,7-tetrasilaoctane |
| 3-Ethylpentan-3-ol |
| 3-Hydroxymethyl-2-trimethylsilyloxypentane |
| 4-((5-Ethenyl-1-azabicyclo(2,2,2)octan-2-yl)oxymethyl)-6-methoxy-quinoline |
| 4,4'-Bitriazolyl |
| 4-Benzyl-1-(1-methyl-2-[(trimethylsilyl)oxy]-2-{4-[(trimethylsilyl)oxy]phenyl}ethyl)piperidine |
| 4H-1,2,4-Triazol-4-amine |
| 4-Hydroxy-5-methyl-1-(tetrahydrofuran-2-yl)pyrimidin-2(1H)-one |
| 4-Pyrimidinamine |
| 5a-Pregnane-3beta,20a-diamine |
| 5F-PB-22 |
| 5-Hydroxy-2-methylpyridine |
| 8-(4-Hydroxyphenyl)-1-methyl-3H,6H,7H-imidazo[1,2-g]purine-2,4-dione |
| 8-Heptadecene |
| Benzenamine |
| Benzo[1,2-c:3,4-c':5,6-c'']tris[1,2,5]oxadiazole |
| Disilane |
| Ethyl pipecolinate |
| Methyl (2R,3R,4S)-3-(tert-butyldimethylsilyloxy)-2,4-dimethylhexanoate |
| Methyl (Z)-10-pentadecenoate |
| Methyl 2,2-dimethyl-3,6,9,12,15-pentaoxa-2-silaheptadecan-17-oate |
| Methyl 2-hydroxy-4-methyl-4-nitroso-pentanoate |
| Methylmalonic monoamide |
| N(1)-[4-[4-Methoxyphenyl]-6-[trichloromethyl]-2-pyrimidinyl]-N(2),N(2)- |
| N,N-Diethylheptylamine |
| N-Acetyl-veratramine |
| N-Dimethylaminomethyl-tert.-butyl-isopropylphosphine |
| N-Ethyldiethanolamine |
| Ortetamine |
| Tetratetracontane |
| Trifluoroacetamide |
| (1-Benzyl-3-phenyl-prop-2-ynyl)-dimethyl-amine |
| (S)-(+)-2-Pyrrolidinemethanol |
| (Z)-Methyl hexadec-11-enoate |
| [2-Pyrrolidinyl]methylamine |
| 1,2,4,5-Tetrazine, 3,6-diphenyl- |
| 1,2-Bis(1,4,7-trioxa-10-azacyclododec-10-yl)-ethane |
| 1-Cyclohexyldimethylsilyloxy-2-phenylethane |
| 1-Cyclohexylethanol |

| |
|---|
| 1-Phenyl-2-(2-trimethylsilylcyclopropen-1-yl)ethanol |
| 2,2,6,6-Tetramethyl-2,6-disilapiperidine |
| 2,3,4-Trifluorobenzoic acid |
| 2,5-Dimethoxy-4-nitrophenethylamine |
| 2,6,10-Trimethyltridecane |
| 2-[[2-[Dimethylamino]propyl]amino-4-[trichloromethyl]-6-[a,a,a-trichloro-m-tolyl]-S-triazine |
| 2-Methyl-2-(trimethylsilyloxy)-1-(4-(2-(trimethylsilyloxy)ethoxy)phenyl)propan-1-one |
| 2-Methyl-8-hydroxyquinoline |
| 2-Pentamethyldisilanyloxybutane |
| 2-Phenyl-1,3-oxazol-2-ine |
| 3,8-Dioxa-2,9-disiladecane |
| 3H-1,2,4-Triazol-3-one, 1,2-dihydro- |
| 3-Methoxyhex-1-ene |
| 3-Methyl-1,3-bis(trimethylsilyloxy)butane |
| 3-Propionyloxytridecane |
| 3-Trimethylsilylmethyl-4-hydroxy-2-methyl-1-hexene |
| 4-Ethyl-4-methyl-1-hexene |
| 4-methoxy-N,N-Dimethylcathinone |
| 5-fluoro PB-22 N-(2-fluoropentyl) isomer |
| 9-Acetylhydrazono-3,6-dichloro-2,7-bis-[2-(diethylamino)ethoxy]fluorene |
| Benzimidazo[2,1-a]isoquinoline |
| Bromosuccinic acid |
| Desethylterbutylazine |
| Ether |
| Etonitazene |
| Methyl 2-hydroxy-2-(4-hydroxyphenyl)propanoate |
| Metoprolol |
| N,O-Bis(tert-butyldimethylsilyl)carbamate |
| N-[beta-Hydroxy-beta-[4-[1-adamantyl-6,8-dichloro]quinolyl]ethyl]piperidine |
| N-Benzhydrylidene-1-(2,4,6-trimethylphenyl)ethylamine N-oxide |
| N-Ethylformamide |
| Propanamide |
| Propanolol |
| Spiro[bicyclo[2.2.1]heptane-2,2'-[1,3]dioxolane]-3-one |
| Tolycaine |
| Trimethylsilyl 3-(3-chloro-4-[(trimethylsilyl)oxy]phenyl)-2-[(trimethylsilyl)amino]propanoate |
| Tris(trimethylsilyl)carbamate |
| (±)-N-Ethylcathinone ephedrine |
| (5-methoxyindol-3-yl)-N,N-diisopropyl-glyoxylamide |
| (Methoxymethyl)trimethylsilane |
| (Z)-5-Methoxy-3,5-dimethyl-2-hexenyltrimethylsilane |
| 1-(2-(3-Cyclohexenyl)ethyl)silatrane |
| 1-(2-Methoxyethoxy)-2-methyl-2-propanol |
| 1-(3,4-Methylenedioxyphenyl)-2-methylamino-1-butanone |
| 1-(3-Methylbutyl)-2,3,4,6-tetramethylbenzene |
| 3-Furoic acid |
| 2-Propenoic acid |
| Scyllo-Inositol |

| |
|---|
| 2-Butyne-1,4-diol |
| 5-Octadecene |
| Eicosane |
| 3-Hexadecene |
| Ethane |
| Nonane |
| Heptane |
| Decane |
| Benzoin |
| 3-Methylpiperazine-2,5-dione |
| Benzene |
| 4-Hydroxybenzoic acid |
| 1-Naphthylamine |
| Phenanthrene |
| Flecainide |
| Phthalic acid |
| Dibutyl phthalate |
| Benzyl thiocyanate |
| Butylone |
| Pentylone |
| 1-Butanone |
| 2,4-Pentanedione |
| 4-Methylethcathinone |
| 2-Butanone |
| 2-Cyclohexen-1-one |
| Octadecanamide |
| Oleamide |
| Cloricromen |
| Cyclohexane |
| Cyclopropane |
| Cyclopropene |
| Cyclohexanamine |
| Mevalonolactone |
| Triethylene glycol |
| 2(3H)-Furanone |
| 2-Furoic Acid |
| Boron |
| 1,3-Butadiyne |
| Melatonin |
| indole-3-acetamide 3 |
| Phthalimide |
| Isoquinolinium |
| 4-(2-Aminoethyl)-2-methoxyphenol |
| Adrenaline |
| Butalamine |
| 1-Hexanamine |
| 3-Amino-2,4-dimethylpentane |
| Diisopropylamine |
| Isopropylamine, N-trimethylsilyl- |
| spermidine |
| Ethylamine |
| Terbutaline |
| (R)-(-)-Phenylephrine |

| |
|---|
| Hydroquinone |
| Piperidine |
| 2-Piperidinone |
| 5-Hydroxymaltol |
| Pyrazine |
| Isonicotinic Acid |
| Picolinic acid |
| 2-hydroxypyridine |
| Pyridine |
| 2(1H)-Pyridinone |
| Cytosine |
| Pyrrole-2-carboxylic acid |
| 1-Methyl-5-mercaptotetrazole |
| Undecene |
| 1-Heptadecene |
| Dodecene |
| N,N'-Dimethylurea |
